# Skin repair and immunoregulatory effects of myeloid suppressor cells from human cord blood in atopic dermatitis

**DOI:** 10.3389/fimmu.2023.1263646

**Published:** 2024-01-09

**Authors:** Chang-Hyun Kim, Seung-Min Hong, Sueon Kim, Jae Ik Yu, Soo-Hyun Jung, Chul Hwan Bang, Ji Hyun Lee, Tai-Gyu Kim

**Affiliations:** ^1^ ViMedier Platform Group, ViGenCell Inc., Seoul, Republic of Korea; ^2^ Department of Dermatology, Seoul St. Mary’s Hospital, College of Medicine, The Catholic University of Korea, Seoul, Republic of Korea; ^3^ Department of Microbiology, College of Medicine, The Catholic University of Korea, Seoul, Republic of Korea

**Keywords:** atopic dermatitis, inflammatory responses, myeloid-derived suppressor cells, skin repair, T-cells

## Abstract

**Introduction:**

Previously, we achieved large-scale expansion of bone marrow-derived suppressor cells (MDSCs) derived from cluster of differentiation (CD)34^+^ cells cultured in human umbilical cord blood (hUCB) and demonstrated their immunomodulatory properties. In the present study, we assessed the therapeutic efficacy of hUCB-MDSCs in atopic dermatitis (AD).

**Methods:**

*Dermatophagoides farinae* (Df)-induced NC/Nga mice (clinical score of 7) were treated with hUCB-MDSCs or a control drug. The mechanisms underlying the therapeutic effects of hUCB-MDSCs were evaluated.

**Results and discussion:**

hUCB-MDSCs demonstrated immunosuppressive effects in both human and mouse CD4^+^ T cells. hUCB-MDSCs significantly reduced the clinical severity scores, which were associated with histopathological changes, and reduced inflammatory cell infiltration, epidermal hyperplasia, and fibrosis. Furthermore, hUCB-MDSCs decreased the serum levels of immunoglobulin E, interleukin (IL)-4, IL-5, IL-13, IL-17, thymus- and activation-regulated chemokines, and thymic stromal lymphopoietin. Additionally, they altered the expression of the skin barrier function-related proteins filaggrin, involucrin, loricrin, cytokeratin 10, and cytokeratin 14 and suppressed the activation of Df-restimulated T-cells via cell–cell interactions. hUCB-MDSCs promoted skin recovery and maintained their therapeutic effect even after recurrence. Consequently, hUCB-MDSC administration improved Df-induced AD-like skin lesions and restored skin barrier function. Our findings support the potential of hUCB-MDSCs as a novel treatment strategy for AD.

## 1 Introduction

Atopic dermatitis (AD) is characterized by complex interactions among genetic, pharmacological, environmental, and psychological factors (1, 2). Its pathogenesis involves systemic immune dysregulation, epidermal barrier dysfunction, and allergen-specific immunoglobulin E (IgE) hypersensitivity; however, the underlying mechanisms remain unclear (3, 4). T-Helper (Th)2 cell differentiation from naïve cluster of differentiation (CD)4^+^ T cells increases interleukin (IL)-4, IL-5, and IL-13 production. Additionally, Th2 cytokines promote mast cell and eosinophil proliferation and activation, thereby increasing IgE level (5). Severe AD may lead to the excessive use of topical or systemic anti-inflammatory and immunosuppressive agents (6, 7), with long-term toxicity and short-term efficacy. Moreover, complete AD suppression is not feasible with recently developed treatments (6–8). Therefore, safer and more effective alternative therapies are needed.

Myeloid-derived suppressor cells (MDSCs) represent a heterogeneous population of hematopoietic cell precursors—macrophages, granulocytes, and dendritic cells—in various maturation states that accumulate in the bone marrow, spleen, and blood (9). MDSCs have garnered attention owing to their increased activity during cancer and inflammatory responses and involvement in tissue damage in an immunosuppressive environment. Their distinctive characteristics are heterogeneous morphology, phenotypes, and functions. Gr-1^+^CD11b^+^ cells in mice and lineage-human leukocyte antigen-DR isotype-low/CD11b^+^CD33^+^ cells in humans are MDSC markers (10). These cells induce immunomodulatory mediators such as indoleamine 2,3-deoxygenase (IDO), inducible nitric oxide synthase (iNOS), and arginase-1 (ARG1) (11, 12). MDSCs inhibit T and NK cell immune responses and promote regulatory T (Treg) cell generation (13, 14). Several receptor–ligand interactions between MDSCs and immune cells regulate immune responses (15). The immunomodulatory and anti-inflammatory properties of these cells have been demonstrated in experimental inflammatory and autoimmune disease models (16–18). Additionally, MDSCs play a role in wound healing (19–21). Among MDSC secretions, transforming growth factor-β and ARG1 contribute to wound healing by producing collagen from fibroblasts (19). Moreover, nitric oxide, generated from inducible nitric oxide synthase (iNOS), contributes to wound healing via several mechanisms (22).

We recently reported that human umbilical cord blood (hUCB)-MDSCs suppress graft-versus-host disease (GVHD) development in mice by selectively suppressing Th2- and Th17-mediated immune responses (23). However, the effects of hUCB-MDSCs and underlying mechanisms in experimental AD mouse models remain to be elucidated. Therefore, in the present study, we investigated the mechanisms underlying the skin-repair and immunomodulatory effects of hUCB-MDSC therapy in a mouse model of *Dermatophagoides farinae* (Df)-induced AD-like skin lesions.

## 2 Materials and methods

### 2.1 Culture of hUCB-MDSCs

**Figure 1:**
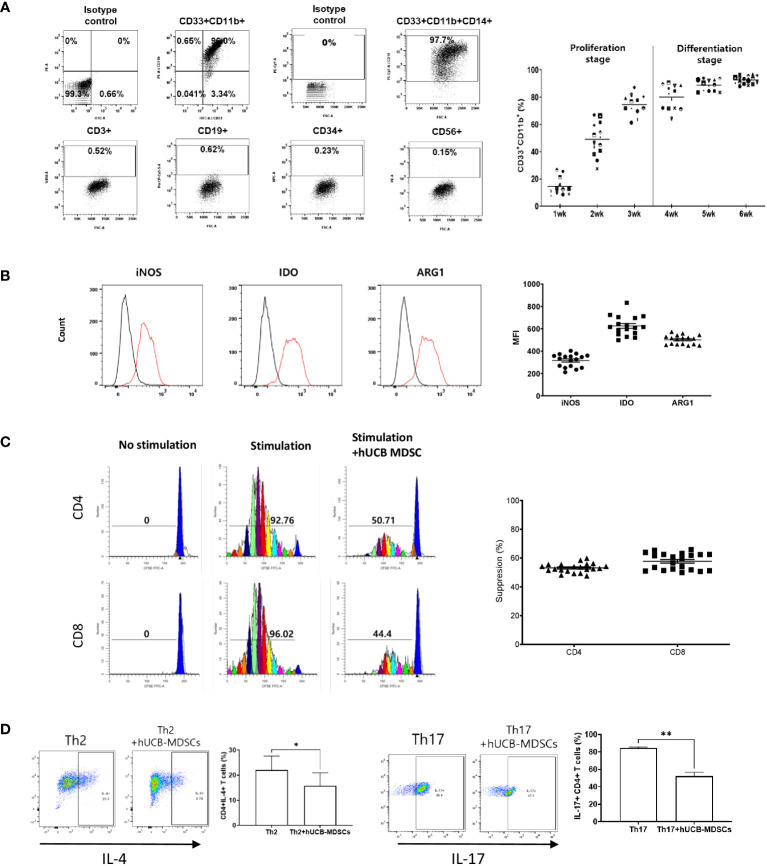
Immunobiological characterization of human umbilical cord blood (hUCB) myeloid-derived suppressor cells (MDSCs) *in vitro* using human T cells. **(A)** The proliferation- and differentiation-stage CD33^+^CD11b^+^ MDSC markers during the 6-week culture period of hUCB-MDSCs. Flow cytometric analysis of hUCB-MDSCs stained with individual MDSC-positive or -negative surface marker antibodies. (CD33^+^CD11b^+^ ≥ 70%, CD14^+^ ≥ 90%) **(B)** The expression of immune suppressive molecules by hUCB-MDSCs was analyzed using FITC anti-inducible nitric oxide synthase (iNOS)2 antibody, PE anti-indoleamine 2,3-deoxygenase (IDO) antibody, and PerCP-Cy5.5 anti-arginase-1 (ARG1) antibody staining. Cells were analyzed using a FACS Lyric device after two washes in stain buffer and incubation on ice for 30 min. Black, isotype control antibody. Red, each specific antibody. (iNOS ≥ 200 MFI, IDO ≥ 450 MFI, ARG1 ≥ 400 MFI) **(C)** hUCB-MDSC-mediated immune suppression of T cells, stimulated by anti-CD3/CD28 beads for 6 days in human peripheral blood mononuclear cells (PBMCs), was tested at a PBMC–hUCB-MDSC ratio of 1:1. (Suppression ≥ 45%). **(D)** hUCB-MDSC-mediated immunoregulation of human CD4^+^ T-cell differentiation, stimulated using Th2 or Th17 differentiation kits for 13 or 6 days in human CD4^+^ T cells, was tested at a human CD4^+^ T cells–hUCB-MDSC ratio of 1:1. *p < 0.05, **p < 0.01.

All experimental procedures using human cord blood derivatives, including hUCB-MDSCs, were conducted following the guidelines approved by the Korea National Institute for bioethics policy (IRB no. P01-202010-31-008). hUCB-MDSCs were isolated and cultured as previously described (**Figure 1A**) (23, 24).

### 2.2 Animals and reagents

Five-week-old female *NC/Nga* mice (Japan SLC, Hamamatsu, Japan) were allowed to acclimatize for 1 week before the experiments. The mice were maintained under specific pathogen-free conditions in the animal care facility of the Catholic University of Korea. The Animal Care and Use Committee of the Research Institute at the Catholic University of Korea approved the experiments (IACUC no.: CUMC-2021-0114-04). Df body ointment was prepared by Biostir, Inc. (Kobe, Japan). One gram of the ointment contained 136.4 mg protein, 234 μg Df 1, and 7 μg Df 2.

### 2.3 *In vitro* T cell assay

Splenocytes from NC/Nga mice were seeded at a density of 1 × 10^6^ cells/well into 24-well plates (Falcon, Corning, NY, USA) and stimulated with CD3/CD28 DYNABEADS (Gibco, Thermo Fisher Scientific, Waltham, MA, USA) to analyze T cell proliferation. hUCB-MDSCs were cocultured with spleen cells at ratios of 0.5:1, 0.25:1, and 0.1:1. Thereafter, cell proliferation was assessed using the Cell Trace CFSE Cell Proliferation Kit (Invitrogen, Thermo Fisher Scientific) per the manufacturer’s instructions.

To analyze T-cell differentiation, purified CD4^+^ T cells from NC/Nga mice were seeded at a density of 3.12 × 10^5^ cells/well into 24-well plates (Falcon) and differentiated using the Th2 differentiation kit (STEMCELL Technologies, Vancouver, Canada) or Th17 differentiation kit (R&D systems, Minneapolis, MN, USA). hUCB-MDSCs were cocultured with CD4^+^ T cells at ratios of 1:1, 0.5:1, and 0.25:1. Th2 and Th17 cell cultures were incubated for 6 and 5 days, respectively, according to the manufacturer’s instructions.

### 2.4 Flow cytometry

Splenocytes or lymph node (LN) cells were harvested and stained with eFluor780-fixable viability dye (eBioscience), Pacific Blue-anti-CD3e (BioLegend, San Diego, CA, USA), PE-cyanine (Cy)7-anti-CD8 (BioLegend), and PerCP-Cy5.5-anti-CD4 (BioLegend) antibodies. After fixation and permeabilization, the cells were stained with PE-anti-IL-13, PE-anti-IL-22, allophycocyanin-anti-IL-5, PE-Cy7-anti-IL-4, Alexa Fluor 488-anti-IL-17A, and BV785-anti-IFN-γ antibodies (BioLegend). The data were analyzed using FlowJo (Tree Star, Ashland, OR, USA).

### 2.5 Induction of AD in NC/Nga mice

AD was induced using Df body ointment (25). The back hair of NC/Nga mice was shaved, and the mice were subjected to skin hair removal treatment (Niclean, Ildong, Korea). The skin barrier was disrupted by treating the shaved dorsal skin and surfaces of both ears with 4% sodium dodecyl sulfate 3 h before the application of Df body ointment (100 mg/mouse). These procedures were repeated two times a week for 4 weeks. After 14 days of the initial Df sensitization, the mice were randomly divided into six groups (n = 5 mice per group): normal (untreated), Df-alone (negative control), Df+Dexa (positive control), Df+hUCB-MDSCs (1 × 10^4^ cells per mouse), Df+hUCB-MDSCs (1 × 10^5^ cells per mouse), and Df+hUCB-MDSCs (1 × 10^6^ cells per mouse). hUCB-MDSCs were intravenously administered to these mice two times a week for 2 weeks. Dexa (2 mg/kg) in water was orally administered to these mice five doses per week for 3 weeks by gavage (oral-zoned needle). Additional information is provided in the **Supplementary Materials and Methods**.

### 2.6 Skin lesion scoring

Erythema/hemorrhage, scarring/dryness, edema, and excoriation/erosion were scored as 0 (none), 1 (mild), 2 (moderate), or 3 (severe) per the observed symptoms. The total skin score was defined as the sum of individual scores (26).

### 2.7 Serum IgE, cytokine, and chemokine assays

Mouse serum was collected 1 week after the final administration of hUCB-MDSCs, and total IgE concentration was measured using an enzyme-linked immunosorbent assay (ELISA) kit (Yamasa, Tokyo, Japan), per the manufacturer’s instructions. IL-4, IL-5, IL-13, IL-17, TSLP, and TARC levels were measured using ELISA kits (R&D Systems).

### 2.8 Histological analyses

The dorsal skin was resected, fixed in 10% formalin solution, and embedded in paraffin. The embedded specimens were then serially sectioned (5 μm) with a microtome (HM 325; Thermo Fisher Scientific) and stained with hematoxylin–eosin to observe the histopathological features or with Masson’s trichrome stain to examine the variable deposition of collagen fibers (blue) and skin fibrosis in the lesioned skin. Mast cells and eosinophils were stained with toluidine blue and Congo red, respectively. Additional information is provided in the **Supplementary Materials and Methods**.

### 2.9 *In vitro* Df restimulation assay

Axillary LNs from normal or Df-induced AD-NC/Nga mice were isolated, and a single-cell suspension was prepared. Cell proliferation was assessed using the Cell Trace CFSE Cell Proliferation Kit (Invitrogen), per the manufacturer’s instructions. LN cells (1 × 10^6^ cells) were stimulated with 1 mg/mL Df in a 24-well flat-bottom microplate at 37°C for 4 days. hUCB-MDSCs (5 × 10^5^ cells) were co-cultured either directly on LN cells or in a Transwell of pore size 0.4 μm (Costar, Corning Inc., Corning, NY, USA). ARG1 (Nor-NOHA 0.5 mM; Selleckchem, Houston, TX, USA) or iNOS inhibitor (1400W 0.1 mM; Medchem Express, Monmouth, NJ, USA) and hUCB-MDSCs was simultaneously treated. After incubation, the cells were harvested for flow cytometry.

### 2.10 Statistical analysis

Data were analyzed for statistical significance using Prism 6.0 (GraphPad Software, San Diego, CA, USA). Unless otherwise indicated, a two-sided unpaired Student’s *t*-test was used to compare two groups, and a one-way analysis of variance with a Dunnett *post-hoc* test was used for multiple group comparisons. The results are presented as mean ± standard deviation. Results with *p* < 0.05 were considered significant.

## 3 Results

### 3.1 *In vitro* immunobiological characterization of hUCB-MDSCs

hUCB-MDSCs cultured with hGM-CSF/hSCF for 6 weeks were immune-positive for CD11b, CD33, and CD14, but negative for CD3, CD19, CD34, and CD56 (**Figure 1A**). The levels of ARG1, IDO, and iNOS, the main factors involved in MDSC-mediated immune suppression, were increased in these cells (**Figure 1B**).

**Figure 2:**
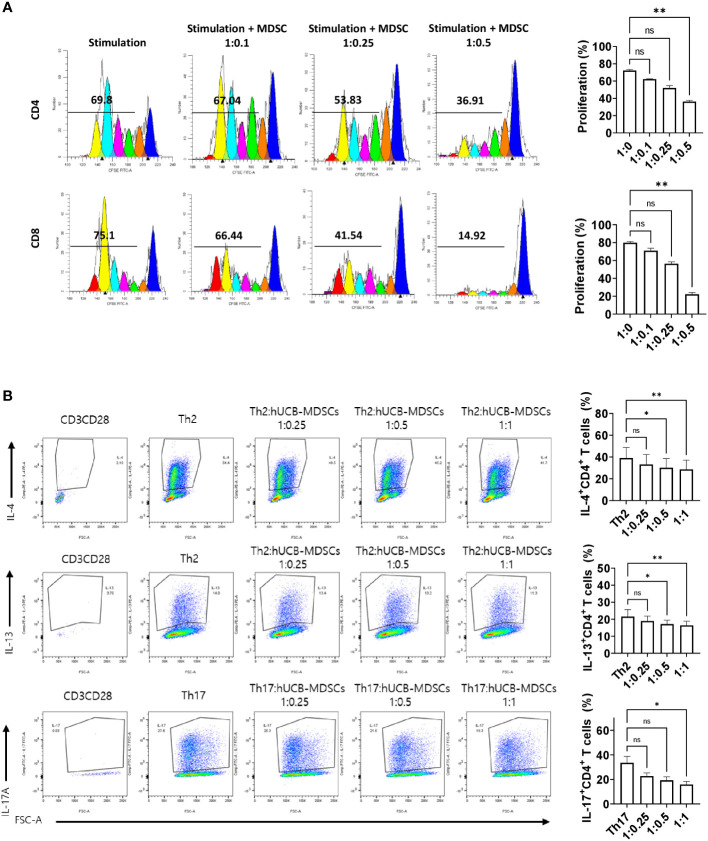
Immunoregulatory capacity of human umbilical cord blood (hUCB) myeloid-derived suppressor cells (MDSCs) to mouse T cells in *in vitro*. **(A)** hUCB-MDSC-mediated immune suppression of T cells, stimulated by anti-CD3/CD28 beads for 4 days, in normal NC/Nga mice, was tested at T-cell–hUCB-MDSC ratios of 1:0, 1:0.1, 1:0.25, and 1:0.5. **(B)** Inhibition of T-helper cell differentiation by hUCB-MDSCs. Naïve CD4^+^ T cells isolated from NC/Nga mice were cocultured with T cell:hUCB-MDSCs (1:0, 1:0.25, 1:0.5, and 1:1 ratios) in the presence of antiCD3, antiCD28, and T cell differentiation kit reagents for 5 or 6 days. Data are presented as mean ± standard error of the mean (n = 3 or 4). *p < 0.05 and **p < 0.01; ns, not significant.

The carboxyfluorescein succinimidyl ester (CFSE) dilution assay confirmed that hUCB-MDSCs effectively inhibited human CD4^+^ and CD8^+^ T cell proliferation (**Figure 1C**). hUCB-MDSCs downregulated the differentiation of human CD4^+^ T cells into Th2 and Th17 cells (**Figure 1D**). Furthermore, they markedly inhibited mouse CD4^+^ and CD8^+^ T cell proliferation stimulated with anti-CD3/CD28 beads at a 1:0.5 (mouse T-cell:hUCB-MDSC) ratio (**Figure 2A**). The addition of hUCB-MDSCs at ratios of 1:1 and/or 1:0.5 to Th2- and Th17-polarizing conditions significantly decreased the IL-4, IL-13, and IL-17 levels compared with those of the untreated group (**Figure 2B**). These results demonstrate the *in vitro* immunosuppressive properties of hUCB-MDSCs against human and mouse T cells.

### 3.2 Migration of hUCB-MDSCs to damaged skin

To investigate the distribution of hUCB-MDSCs, PKH26-labeled hUCB-MDSCs were injected into both atopic and normal control mice and subsequently identified in various organs. Notably, although hUCB-MDSCs were observed in the lungs, spleen, and LNs of both groups, infiltration of hUCB-MDSCs in skin tissue was found only in atopic dermatitis mice (**Supplementary Figure S1**).

### 3.3 Effects of hUCB-MDSCs on Df-induced AD-like skin lesions

**Figure 3:**
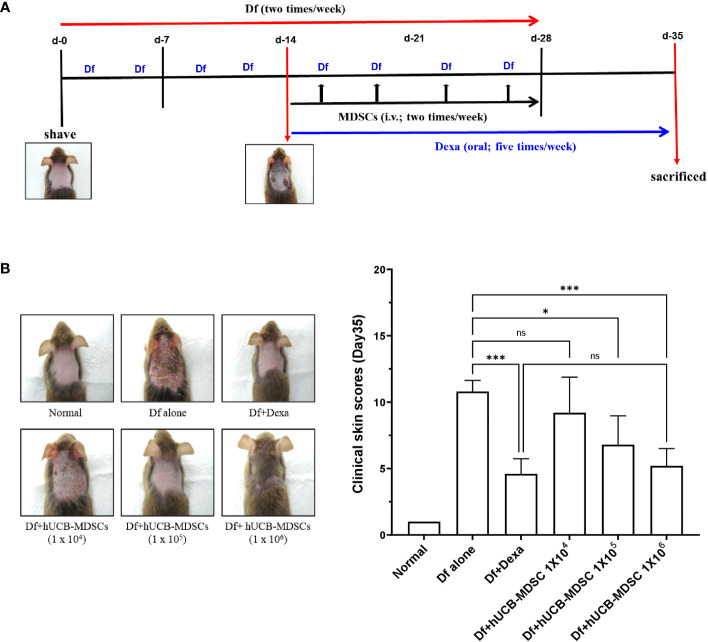
Effects of human umbilical cord blood (hUCB)-myeloid-derived suppressor cell (MDSC) therapy against *Dermatophagoides farinae* (Df)-induced atopic dermatitis skin score. **(A)** Schematic diagram of the animal experiments. After hair removal, Df body ointment (100 mg/mouse) was swabbed on the dorsal skin and surfaces of both ears twice a week for 4 weeks. Fourteen days after the first sensitization, Df-induced NC/Nga mice were either not treated (negative control; Df-alone) or treated with hUCB-MDSCs (1 × 10^4^, 1 × 10^5^, or 1 × 10^6^ cells/mouse) intravenously twice a week for 2 weeks. Dexamethasone (Dexa) (positive control; 2 mg/kg) was orally administered in five doses per week for 3 weeks. **(B)** Images of the skin of five mice (left). Clinical skin scores of mice are presented as the sum of individual scores of erythema/hemorrhage, scarring/dryness, edema, and excoriation/erosion (right). *p < 0.05, ***p < 0.001; ns, not significant.

**Figure 4:**
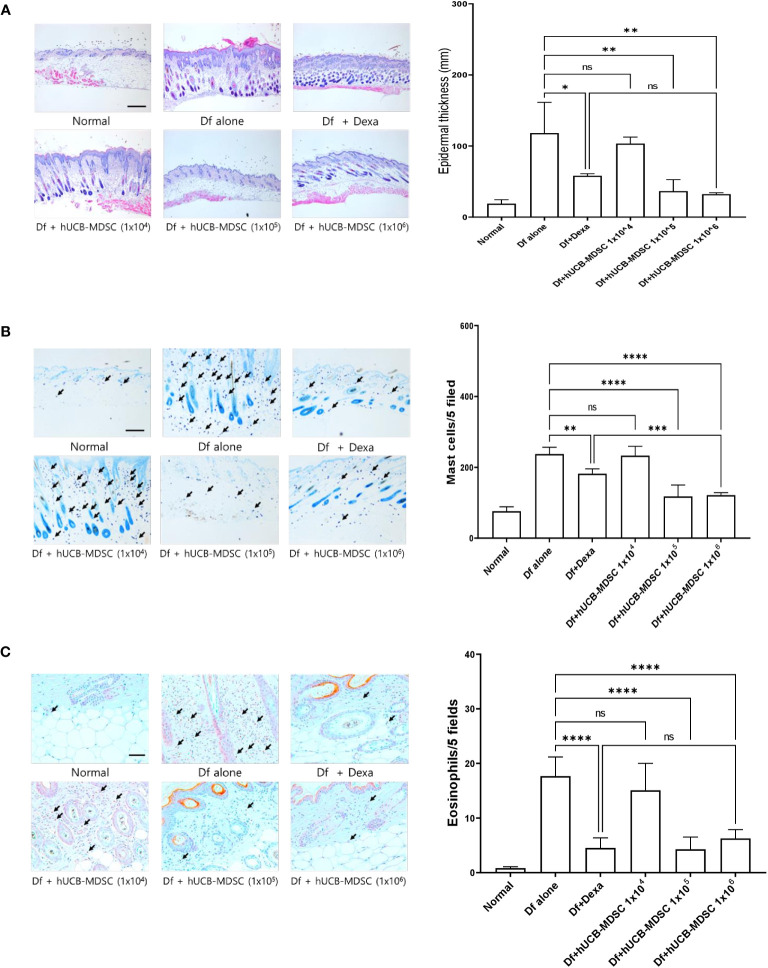
Effects of human umbilical cord blood (hUCB)-myeloid-derived suppressor cell (MDSC) therapy against *Dermatophagoides farinae* (Df)-induced atopic dermatitis-like skin lesions. **(A)** Hematoxylin- and eosin-stained sections showing epidermal thickness (hyperplasia) at five randomized sites under 100× magnification. **(B, C)** Sections were stained with toluidine blue and Congo red for visualizing mast cells and eosinophils, respectively. The cell count is expressed as the number of mast cells and eosinophils in five high-power fields (×400 for the count) for each section. Data are presented as mean ± standard error of the mean of six mice per group in a representative experiment out of two experiments. Scale bar = 400 µm **(A)**, 200 µm **(B)** and 50 µm **(C)**. *p < 0.05, **p < 0.01, ***p < 0.001, and ****p < 0.0001; ns, not significant.

The schematic diagram of the experiment is illustrated in **Figure 3A**. Dexamethasone (Dexa), an anti-inflammatory and immunosuppressive drug used for treating AD, served as a positive control. The symptom severity scores of the Df+hUCB-MDSC (1 × 10^5^ and 1 × 10^6^ cells) and Df+Dexa groups were considerably improved compared with those of the Df-alone group (**Figure 3B**), whereas no difference was observed compared with those of the Df+hUCB-MDSC (1 × 10^4^ cells) group. When hUCB-MDSCs were injected once or twice, the effect of hUCB-MDSCs (1 × 10^5^ and 1 × 10^6^ cells) was not observed (**Supplementary Figure S2**). Df-induced skin inflammation in mice presented as epidermal hyperplasia, hyperkeratosis, and lymphocyte infiltration into the epidermis and dermis, which are typical histopathological characteristics of human AD (2, 3). The Df+hUCB-MDSC (1 × 10^5^ and 1×10^6^ cells) and Df+Dexa groups exhibited amelioration of histopathological characteristics (**Figure 4A**). Epidermal thickness considerably decreased in the Df+hUCB-MDSC and Df+Dexa groups compared with that in the Df-alone group (**Figure 4A**). Treatment with Df+hUCB-MDSCs (1 × 10^5^ and 1 × 10^6^ cells) led to a significant decrease in the number of infiltrating mast cells compared with that in the Df+Dexa and Df-alone groups (**Figure 4B**). Furthermore, the extent of decrease in eosinophil counts following Df+hUCB-MDSC treatment was similar to that observed in the Df+Dexa group (**Figure 4C**). Remarkably, even in a Df-stimulated AD animal model under relapse conditions, hUCB-MDSCs exhibited sustained therapeutic efficacy, in contrast to Dexa which rapidly lost its effectiveness, as confirmed by clinical skin scores (**Supplementary Figure S3**).

### 3.4 Effects of hUCB-MDSCs on skin barrier repair and skin fibrosis

**Figure 5:**
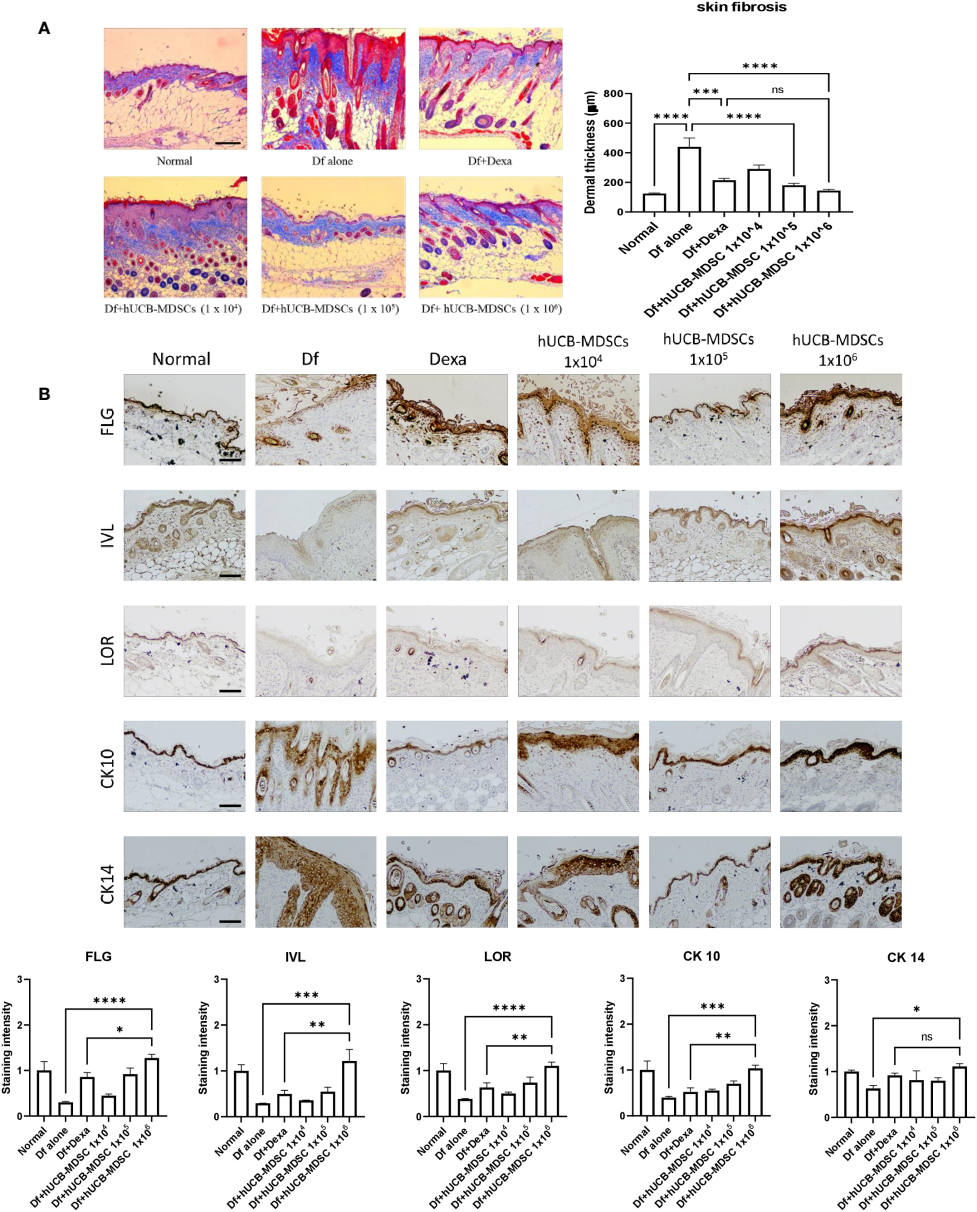
Effects of human umbilical cord blood (hUCB)-myeloid-derived suppressor cell (MDSC) therapy on skin barrier repair and skin fibrosis in *Dermatophagoides farinae* (Df)-induced atopic dermatitis-like skin lesions in NC/Nga mice. **(A)** Masson’s trichrome staining (representative images) (left) and dermal thickness (right). **(B)** Immunohistochemical staining of filaggrin (FLG), involucrin (IVL), loricrin (LOR), keratin-10 (CK10), and keratin-14 (CK14) in skin equivalents from NC/Nga mice not treated (Df alone) or treated with hUCB-MDSCs (1 × 10^4^ cells), hUCB-MDSCs (1 × 10^5^ cells), hUCB-MDSCs (1 × 10^6^ cells), or dexamethasone (Dexa) (2 mg/kg) on day 35. Data are presented as mean ± standard error of the mean of five mice per group in a representative experiment of two experiments. Scale bar = 200 µm **(A, B)**. *p < 0.05, **p < 0.01, ***p < 0.001; ****p < 0.0001; ns, not significant.

Impaired skin barrier function and skin fibrosis are important targets for AD treatment (3, 27). The proportion of dermal collagen matrix considerably increased in the Df-alone group compared with that in the normal group. However, compared with the Df-alone group, the Df+hUCB-MDSC (1 × 10^5^ and 1 × 10^6^ cells) and Df+Dexa groups showed a markedly reduced proportion of dermal collagen matrix (**Figure 5A**). In both Dexa and hUCB-MDSC groups, the epidermal protein levels were higher than those in the Df-alone group (**Figure 5B**). Notably, the levels of epidermal proteins such as FLG, IVL, LOR, and CK10 were significantly higher in the Df+hUCB-MDSC (1 × 10^6^ cells) group than in the positive control group treated with Dexa (**Figure 5B**). Overall, hUCB-MDSCs display potential as an alternative treatment strategy to avoid the adverse effects of Dexa-associated skin atrophy.

### 3.5 Effects of hUCB-MDSCs on increased levels of IgE and inflammatory mediators

**Figure 6:**
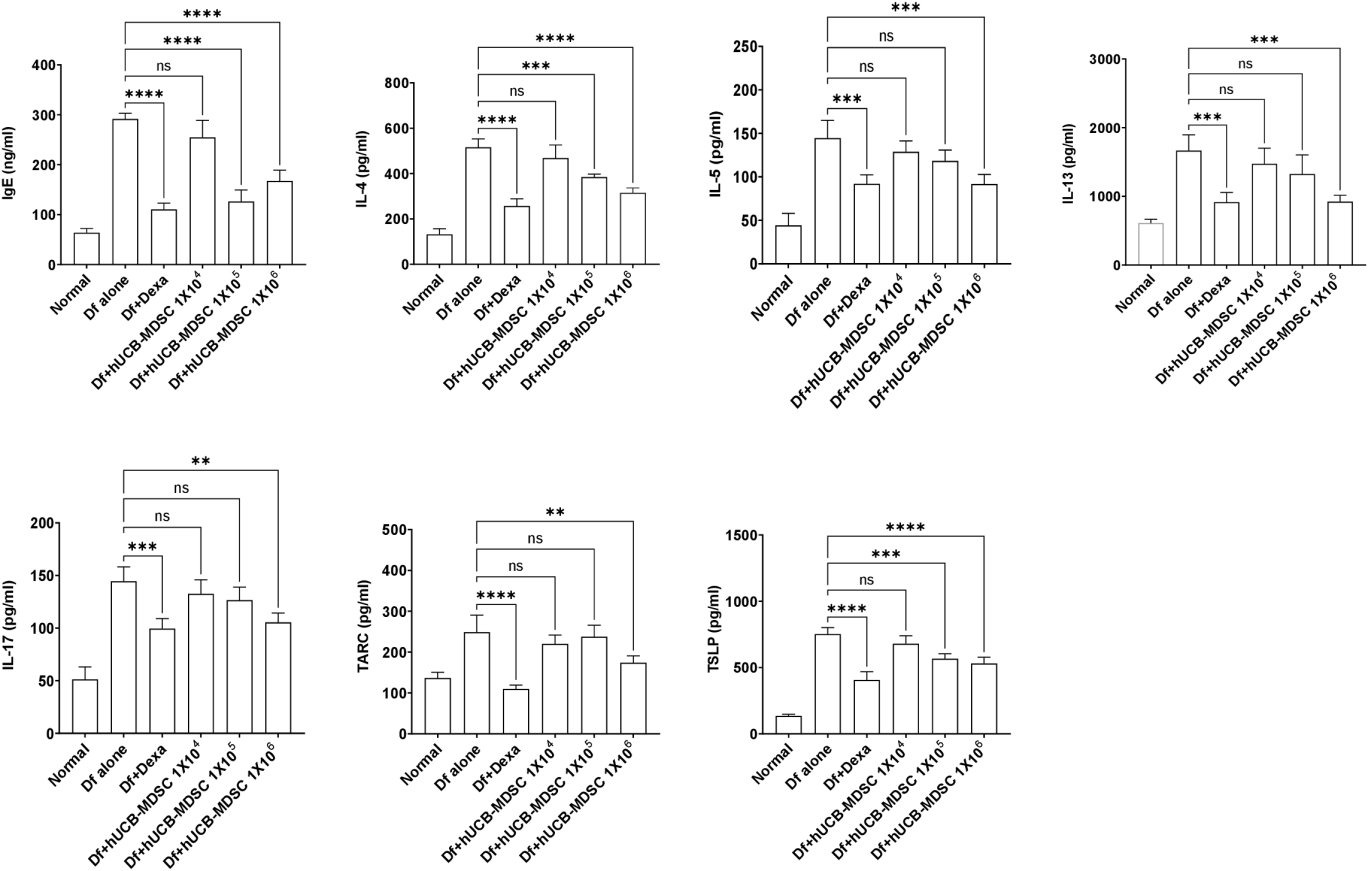
Effects of human umbilical cord blood-myeloid-derived suppressor cell therapy on the levels of IgE and inflammatory mediators in the serum of *Dermatophagoides farina* (Df)-induced NC/Nga mice. IgE, IL-4, IL-5, IL-13, IL-17, TARC, and TSLP levels were measured on day 7 after the final administration of hUCB-MDSCs. Data are presented as mean ± standard error of the mean of five mice per group in a representative experiment of two experiments. **p < 0.01, ***p < 0.001, and ****p < 0.0001; ns, not significant.

The total serum IgE level was considerably lower in the Df+hUCB-MDSC (1 × 10^5^ and 1 × 10^6^ cells) and Df+Dexa groups than in the Df-alone group; however, no notable difference was observed between the Df+hUCB-MDSC (1 × 10^4^ cells) and Df-alone groups (**Figure 6**). The Th2 (IL-4, IL-5, and IL-13) and Th17 cytokine (IL-17) levels markedly decreased in the Df+hUCB-MDSC (1 × 10^5^ and/or 1 × 10^6^ cells) and Df+Dexa groups compared with those in the Df-alone group. Moreover, the serum thymic stromal lymphopoietin (TSLP) and thymus- and activation-regulated chemokine (TARC) levels considerably decreased in the Df+hUCB-MDSC (1 × 10^5^ and/or 1 × 10^6^ cells) and Df+Dexa groups. Conversely, there was no considerable difference between the Df+hUCB-MDSC (1 × 10^4^ cells) and Df-alone groups in terms of TSLP and TARC levels.

### 3.6 Effects of hUCB-MDSCs on the regulation of differentiation to CD4+ T-cell subsets

**Figure 7:**
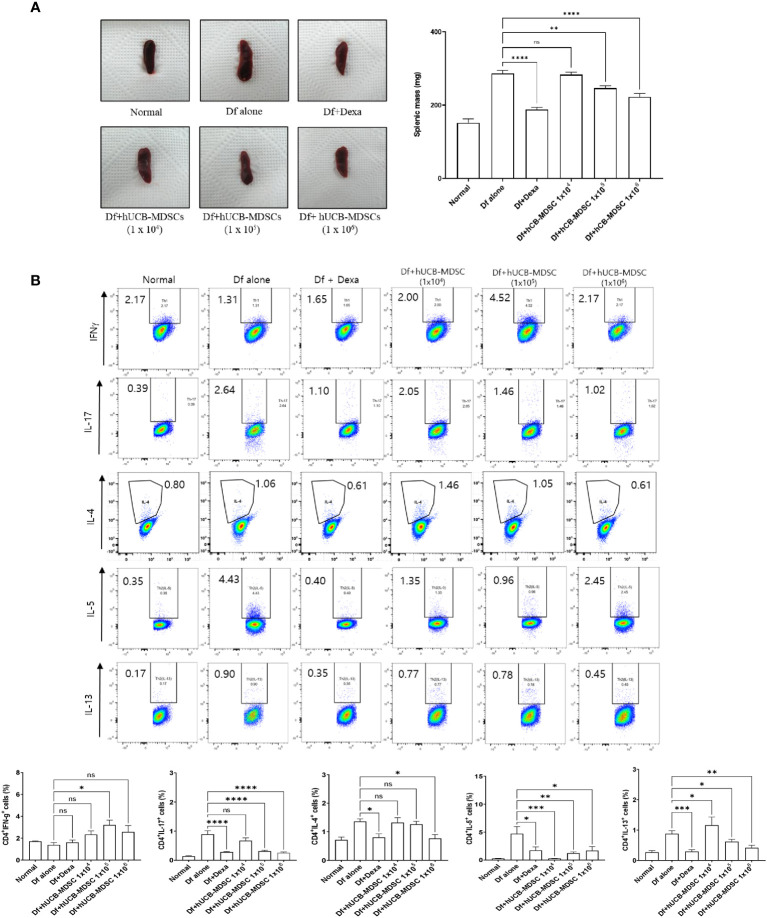
Effects of human umbilical cord blood (hUCB)-myeloid-derived suppressor cell (MDSC) therapy on the regulation of differentiation to CD4^+^ T cell subsets. **(A)** Representative photograph of the spleen collected from untreated and treated NC/Nga mice; mice were treated with *Dermatophagoides farinae* (Df) alone, hUCB-MDSCs (1 × 10^4^ cells), hUCB-MDSCs (1 × 10^5^ cells), hUCB-MDSCs (1 × 10^6^ cells), or dexamethasone (Dexa) (2 mg/kg). Spleen weights of mice sacrificed on day 7 after the final administration of hUCB-MDSCs. **(B)** Flow cytometric analysis illustrating T-helper (Th)1 cells (cluster of differentiation [CD]4^+^ interferon-γ^+^), Th2 cells (CD4^+^ interleukin [IL]-4^+^, CD4^+^ IL-5^+^, and CD4^+^ IL-13^+^), and Th17 cells (CD4^+^ IL-17^+^) in the splenocytes of NC/Nga mice. The data are presented as mean ± standard error of the mean of five mice per group in a representative experiment of two experiments. *p < 0.05, **p < 0.01, ***p < 0.001, and ****p < 0.0001; ns, not significant.

The spleen weight and size of the Df-alone group were notably higher than those of the normal control group. Compared with the Df-alone group, the Df+hUCB-MDSC (1 × 10^5^ and 1 × 10^6^ cells) and Df+Dexa groups showed considerably reduced spleen weight and size (**Figure 7A**). Analysis of effector CD4^+^ T cell subsets in the mouse spleen revealed that the number of IL-4-, IL-5-, IL-13-, and IL-17-producing CD4^+^ T cells in the splenocytes of Df-induced NC/Nga mice was notably lower in the Df+hUCB-MDSC (1 × 10^5^ cells and/or 1 × 10^6^ cells) group than in the Df+hUCB-MDSC (1 × 10^4^ cells) and Df-alone groups (**Figure 7B**). Additionally, IL-5 production was decreased in the Df+hUCB-MDSC (1 × 10^4^ cells) group, whereas IFN-γ production was increased in the Df+hUCB-MDSC (1 × 10^5^ cells) group.

The proportion changes of effector CD4^+^ T cell subsets caused by hUCB-MDSCs in the LNs are illustrated in **Supplementary Figure S4**. The number of IL-4-, IL-5-, IL-13-, and IL-17-producing CD4^+^ T cells in the LNs of Df-induced NC/Nga mice was decreased in the Df+hUCB-MDSC (1 × 10^5^ cells and 1 × 10^6^ cells) and/or Df+hUCB-MDSC (1 × 10^4^ cells) groups, whereas IFN-γ production was increased in the Df+hUCB-MDSC (1 × 10^5^ cells and 1 × 10^6^ cells) groups.

### 3.7 Immune regulatory mechanism between hUCB-MDSCs and T cells

**Figure 8:**
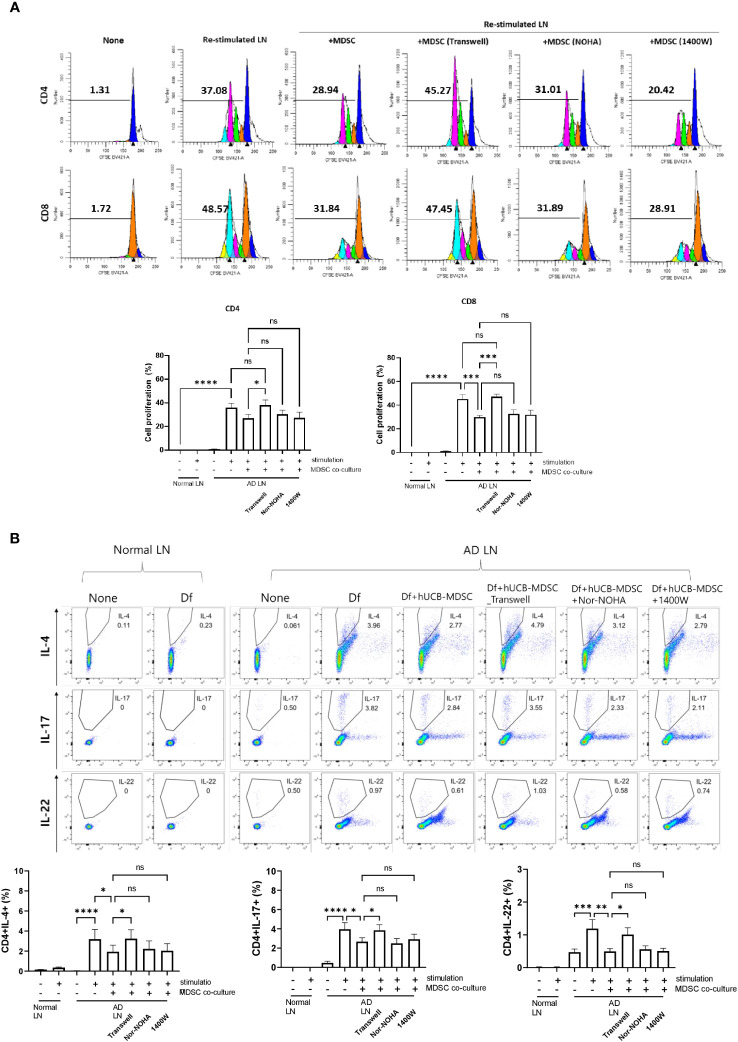
Mechanism of atopic dermatitis (AD)-associated immune cell regulation between human umbilical cord blood (hUCB)-myeloid-derived suppressor cells (MDSCs) and T cells in the AD mouse model. Lymph node (LN) cells from Nc/Nga mice were stimulated with treatments for 4 days *in vitro*. **(A)** Representative experimental data demonstrating the loss of the ability of hUCB-MDSCs to suppress T cell stimulation in the Transwell assay. The intensity of CFSE-labeled T cells was acquired using flow cytometry and further analyzed using ModFit LT 4.0 software. **(B)** Flow cytometric analysis of T-helper (Th)2 cells (cluster of differentiation [CD]4^+^ interleukin [IL]-4^+^), Th17 cells (CD4^+^ IL-17^+^), and Th22 cells (CD4^+^ IL-22^+^). Data are presented as mean ± standard error of the mean of three to nine mice per group. *p < 0.05, **p < 0.01, ***p < 0.001 and ****p < 0.0001; ns, not significant.

Restimulation of lymph node cells collected from mice with Df-induced AD increased the proliferation of CD4^+^ and CD8^+^ T cells, and hUCB-MDSCs downregulated the proliferation (**Figure 8A**). However, the Transwell assay revealed that hUCB-MDSCs no longer exhibited immunomodulatory functions in the context of blocked cell-cell interactions, suggesting that secreted immunosuppressive substances had little effect on AD mice (**Figure 8A**). We validated our results using iNOS and ARG1 inhibitors (Nor-NOHA, 1400W), which also did not affect the immunosuppressive function of hUCB-MDSCs (**Figure 8A**). The same results were observed in a subset of CD4^+^ T cells (**Figure 8B**). These findings suggest that the suppressive function of hUCB-MDSCs in AD is primarily dependent on cell–cell interactions rather than on secreted immunosuppressive substances.

## 4 Discussion

MDSCs inhibit T-cell-mediated inflammatory responses via various mechanisms, which may alleviate the disease. However, their small population limits their clinical applications. hUCB can differentiate to immunomodulatory cells such as mesenchymal stem cells (MSCs) and MDSCs (28, 29). Our MDSCs produced at a large scale from hUCB-CD34^+^ cells suppressed the pathological features of GVHD in preclinical models by modulating T-cell-mediated immunity (23, 24).

In our mouse model, the lesioned skin exhibited erythema, scaling, thickening, and inflammatory cell infiltration into the dermis and epidermis, similar to human AD symptoms (2, 3). Inflammatory skin diseases are accompanied by epidermal hyperproliferation and inflammatory cell infiltration into the dermis and epidermis. Here, hUCB-MDSCs alleviated the overall skin lesion severity in a dose-dependent manner; however, a low dose of hUCB-MDSCs (1 × 10^4^ cells) was not effective. Furthermore, hUCB-MDSCs (1 × 10^5^ and 1 × 10^6^ cells) reduced the epidermal thickness of skin lesions and attenuated the infiltration of inflammatory cells.

Similar to the findings of a previous study (23), viable hUCB-MDSCs administered to AD-induced mice were found in the spleen, skin, lungs, and LNs. The lungs are the most common site of invasion for intravenously injected cells, which also migrate to the spleen and lymph nodes (30–34). Therefore, hUCB-MDSCs may have infiltrated the lungs, spleens, and LNs of normal healthy mice. However, infiltration into the skin was found only in AD mice, suggesting that hUCB-MDSCs selectively infiltrated sites of inflammation (35, 36). In the future, research on chemotaxis related to these movements should be conducted.

AD is associated with skin fibrosis and barrier disruption of the stratum spinosum, stratum basale, and stratum granulosum because of epidermal protein function loss (27, 37). Skin barrier dysfunction is associated with AD-related proinflammatory mediator production in mice and patients with AD (38, 39). The levels of various AD-related proinflammatory cytokines increase in patients with AD, leading to defects in skin barrier functions by decreasing epidermal differentiation marker levels (40, 41). The skin barrier integrity of epidermal structures can be disrupted in the stratum basale and stratum spinosum of AD-lesional skin, along with decreased levels of CK10 and CK14, markers for keratinocyte differentiation (42, 43). Skin fibrosis probably results from abnormal repair in response to skin damage, which may be caused by allergic inflammatory responses (44). Skin barrier normalization is related to wound healing (45), and MDSCs are involved in wound healing. Here, hUCB-MDSCs (1 × 10^5^ and/or 1 × 10^6^ cells) restored skin barrier function and improved skin fibrosis, suggesting that the anti-inflammatory effects and wound-healing capacities of hUCB-MDSC therapy are the mechanisms responsible for recovery from skin barrier impairment and dysfunction and skin fibrosis in Df-induced AD-NC/Nga mice.

The activation and overproduction of proinflammatory cytokines are observed in the serum of patients with AD, similar to that in the serum of Df-induced AD mice (46–48), and they are associated with major immunological and cellular mechanisms in AD. Consistently, we observed that the IgE and Th2- and Th17-mediated cytokine levels were increased in the serum of Df-induced NC/Nga mice; however, hUCB-MDSC administration (1 × 10^5^ and/or 1 × 10^6^ cells) considerably suppressed their production. TSLP, TARC, and Th2-specific chemokines in the serum of mice and patients with AD are involved in Th2-mediated immune responses while playing important roles in AD (49–51). TSLP is involved in inflammatory cell (mast cells and eosinophils) proliferation and activation and induces IgE (52). Our results showed that hUCB-MDSCs (1 × 10^5^ and/or 1 × 10^6^ cells) significantly inhibited serum TSLP and TARC production. Therefore, hUCB-MDSCs can alleviate clinical symptoms and T-cell-mediated immune responses by suppressing the production of IgE, several pro-inflammatory cytokines, and chemokines involved in AD.

MDSCs regulate immune cell functions and inflammatory mediators in various autoimmune diseases. Our experiments showed that hUCB-MDSCs inhibit CD4^+^ and CD8^+^ T cell proliferation and Th2 and Th17 differentiation, consistent with the findings of a previous study (12). Additionally, hUCB-MDSCs attenuated Df-induced splenomegaly. These findings suggest that hUCB-MDSC therapy exerts an anti-inflammatory effect by altering the T cell proliferation rate and Th2 and Th17 cell ratio. However, the expression of IFN-γ in the spleen and LN cells was increased by hUCB-MDSCs. IFN-γ is known to exhibit therapeutic effects in AD (53, 54). Moreover, IFN-γ has the potential to enhance the immunomodulatory capabilities of MDSCs (55, 56). However, it is crucial to note that mouse-derived IFN-γ cannot bind to the human IFN-γ receptor, rendering it impossible for mouse IFN-γ to affect human MDSCs (57). Therefore, further studies are necessary to elucidate the role of IFN-γ induced by hUCB-MDSCs in AD mice.

The *in vitro* Df restimulation experiment indicated the important role of interactions between hUCB-MDSCs and immune cells, and not the secreted factors arginase-1 and iNOS, in immunomodulatory actions. Further research on these immunomodulatory mechanisms is needed.

Numerous systemic immunomodulatory agents are used to treat AD. However, the current treatments for severe AD are not always effective. Additionally, basic and preclinical studies using AD models have demonstrated the immunomodulatory and anti-inflammatory effects of MSCs derived from hUCB, bone marrow, and adipose tissues (58). MDSCs developed here were 4–8 times smaller than MSCs, which is advantageous for dosage administration and is potentially safer than MSCs. Moreover, hUCB-MDSCs were intravenously injected, and the medium dose (1 × 10^5^ cells) of hUCB-MDSCs considerably reduced AD allergic progression in mice as effectively as the high dose (1 × 10^6^ cells). These findings suggest that MDSCs, at lower doses, can effectively treat AD.

Our study demonstrated that hUCB-MDSCs alleviated AD-like clinical symptoms by regulating interactions between several upstream and downstream immunological factors involved in progressive AD pathology. hUCB-MDSC therapy exerts immunomodulatory and anti-inflammatory effects with reduced toxicity, suppresses adverse effects, and promotes skin regeneration; therefore, it may be a novel cell therapy for AD.

## Data availability statement

The original contributions presented in the study are included in the article/**Supplementary Material**, further inquiries can be directed to the corresponding author.

## Ethics statement

The studies involving humans were approved by Korea National Institute for bioethics policy. The studies were conducted in accordance with the local legislation and institutional requirements. The participants provided their written informed consent to participate in this study. The animal study was approved by The Animal Care and Use Committee of the Research Institute at the Catholic University of Korea. The study was conducted in accordance with the local legislation and institutional requirements.

## Author contributions

C-HK: Conceptualization, Investigation, Writing – original draft. S-MH: Conceptualization, Investigation, Writing – original draft, Writing – review & editing. SK: Conceptualization, Writing – original draft, Writing – review & editing. JY: Investigation, Writing – original draft. S-HJ: Investigation, Writing – original draft. CB: Conceptualization, Writing – original draft. JL: Conceptualization, Writing – original draft. T-GK: Conceptualization, Project administration, Supervision, Writing – original draft, Writing – review & editing.
