# Supplementary material for: Skin repair and immunoregulatory effects of myeloid suppressor cells from human cord blood in atopic dermatitis
Source: Front Immunol. 2024 Jan 9;14:1263646. doi: 10.3389/fimmu.2023.1263646 (PMC10803405; doi:10.3389/fimmu.2023.1263646)
Supplement: Supplementary file 1 [file DataSheet_1.pdf]

Supplementary Material

Supplementary Materials and Methods

Induction of AD in NC/Nga Mice

To determine the therapeutic efficacy based on the number of injections, after hair removal, Df body ointment (100 mg/mouse) was swabbed on the dorsal skin and the surfaces of both ears two times a week for 4 weeks. Fourteen days after the first sensitization, Df-induced NC/Nga mice were either not treated (negative control; Df-alone) or treated with hUCB-MDSCs (1×10^4, 1×10^5, or 1×10^6 cells/mouse) intravenously once a week for 1 week or once a week for 2 weeks.

To elucidate the persistency of the therapeutic effect, after hair removal, Df body ointment (100 mg/mouse) was swabbed on the dorsal skin and surfaces of both ears two times a week for 4 weeks. Fourteen days after the first sensitization, Df-induced NC/Nga mice were not treated (negative control; Df-alone), treated with hUCB-MDSCs (1×10^4, 1×10^5, or 1×10^6 cells/mouse) intravenously two times a week for 2 weeks, or treated with Dexa (positive control; 0.1% in phosphate-buffered saline [PBS], 200 μL) topically five times per week for 2 weeks. On day 49, Df body ointment was swabbed on the dorsal skin and the surfaces of both ears two times a week for 1 week.

In Vivo Tracking of hUCB-MDSCs

To identify hUCB-MDSCs in the target organs, hUCB-MDSCs were labeled with PKH-26 (Sigma-Aldrich) per the manufacturer's instructions and injected into recipient mice (untreated or 4-week-old Df-treated NC/Nga mice). Tissues were embedded 2 hours after cell injection in the embedding matrix of optimal cutting temperature compound (CellPath, Newtown, Powys, UK), placed on dry ice, and stored at −80°C. Thereafter, they were sectioned with a cryostat (5 mm in thickness), fixed with acetone, washed, and stained with 4,6-diamidino-2-phenylin-dole (Sigma-Aldrich). The slides were examined under a confocal microscope (LSM800; Carl Zeiss, Oberkochen, Germany).

Histological Analyses

To quantify epidermal proteins, deparaffinized sections were incubated in 0.01 M sodium citrate buffer (pH 6.5) for 10 min at 100°C and cooled at 25°C for 10 min. The sections were then treated with 3% hydrogen peroxide in PBS for 15 min at 20°C–22°C to block endogenous peroxidase activity. After blocking the sections with 10% normal goat serum for 1 h at 25°C, they were incubated overnight with FLG, IVL, LOR, cytokeratin 10, or cytokeratin 14. After extensively washing the slides with PBS, they were incubated with reagents of the VECTASTAIN® ABC-HRP Kit (Vector Laboratories, Inc, Burlingame, CA, USA). The sections were counterstained with diaminobenzidine (DAB). The intensity of DAB on the epidermis was quantitatively evaluated using ImageJ (NIH, Bethesda, Maryland, USA).

Supplementary Figures

Supplementary Figure 1. Specific migration of human umbilical cord blood (hUCB)-myeloid-derived suppressor cells (MDSCs) into the injured skin.

Representative confocal images of hUCB-MDSCs in the lung, skin, and secondary lymphoid organs after injection into the tail vein. Data are representative of three mice per group. Scale bar = 20 μm.

Supplementary Figure 2. Therapeutic efficacy based on the number of injections of human umbilical cord blood (hUCB)-myeloid-derived suppressor cells (MDSCs) against *Dermatophagoides farinae* (Df)-induced atopic dermatitis.

Clinical skin scores of mice were presented as the sum of individual scores of erythema/hemorrhage, scarring/dryness, edema, and excoriation/erosion. Data are presented as mean ± standard error of the mean of five mice per group.

Supplementary Figure 3. Maintenance of the therapeutic effect of human umbilical cord blood (hUCB)-myeloid-derived suppressor cells (MDSCs) and rapid reduction in the efficacy of dexamethasone (Dexa) upon restimulation of atopic dermatitis (AD) model with *Dermatophagoides farinae* (Df).

Clinical skin scores of mice were presented as the sum of individual scores of erythema/hemorrhage, scarring/dryness, edema, and excoriation/erosion. Data are presented as mean ± standard error of the mean of five mice per group. Blue asterisk and ns are between Df alone and Df+Dexa. Red asterisk is between Df alone and Df+hUCB-MDSCs(1×10^5 cells per mouse). $^{**}p < 0.01$, $^{***}p < 0.001$, and $^{****}p < 0.0001$; ns, not significant for the two-way analysis of variance followed by Bonferroni post-test.

Supplementary Figure 4. Effects of human umbilical cord blood (hUCB)-myeloid-derived suppressor cell (MDSC) therapy on axillary lymph nodes (LNs).

Flow cytometric analysis showing T-helper (Th)1 cells (cluster of differentiation [CD]4$^+$ interferon-γ$^+$), Th2 cells (CD4$^+$ interleukin [IL]-4$^+$, CD4$^+$ IL-5$^+$, and CD4$^+$ IL-13$^+$), and Th17 cells (CD4$^+$ IL-17$^+$) in the LNs of NC/Nga mice treated with hUCB-MDSCs. The data are presented as mean ± standard error of the mean of five mice per group in a representative experiment of two experiments. $^*p < 0.05$, $^{**}p < 0.01$, $^{***}p < 0.001$, and $^{****}p < 0.0001$; ns, not significant.
