# Supplementary figures and images for: Skin repair and immunoregulatory effects of myeloid suppressor cells from human cord blood in atopic dermatitis
Source: Front Immunol. 2024 Jan 9;14:1263646. doi: 10.3389/fimmu.2023.1263646 (PMC10803405; doi:10.3389/fimmu.2023.1263646)

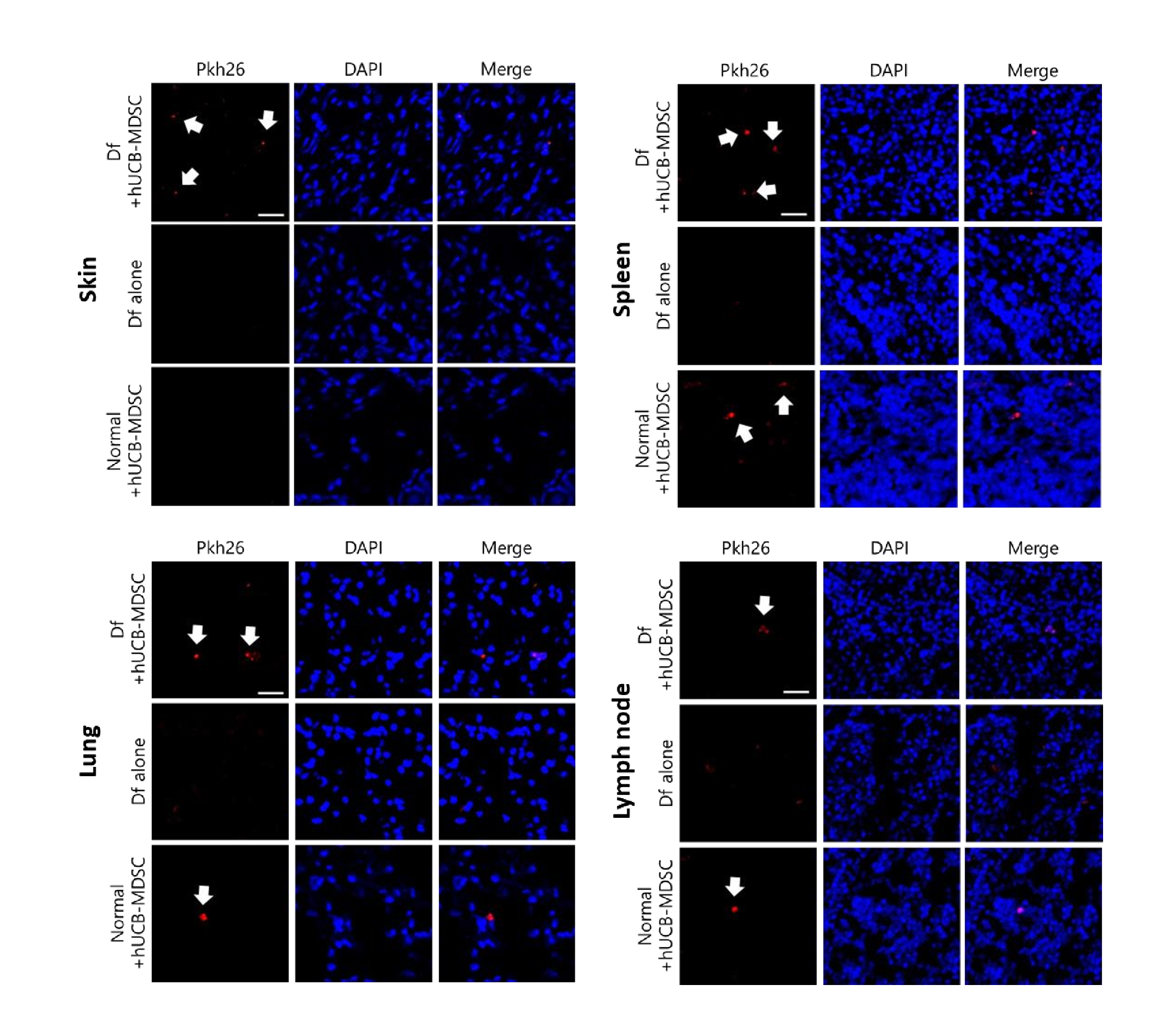

Supplement: Supplementary file 2 [file Image_1.tiff]

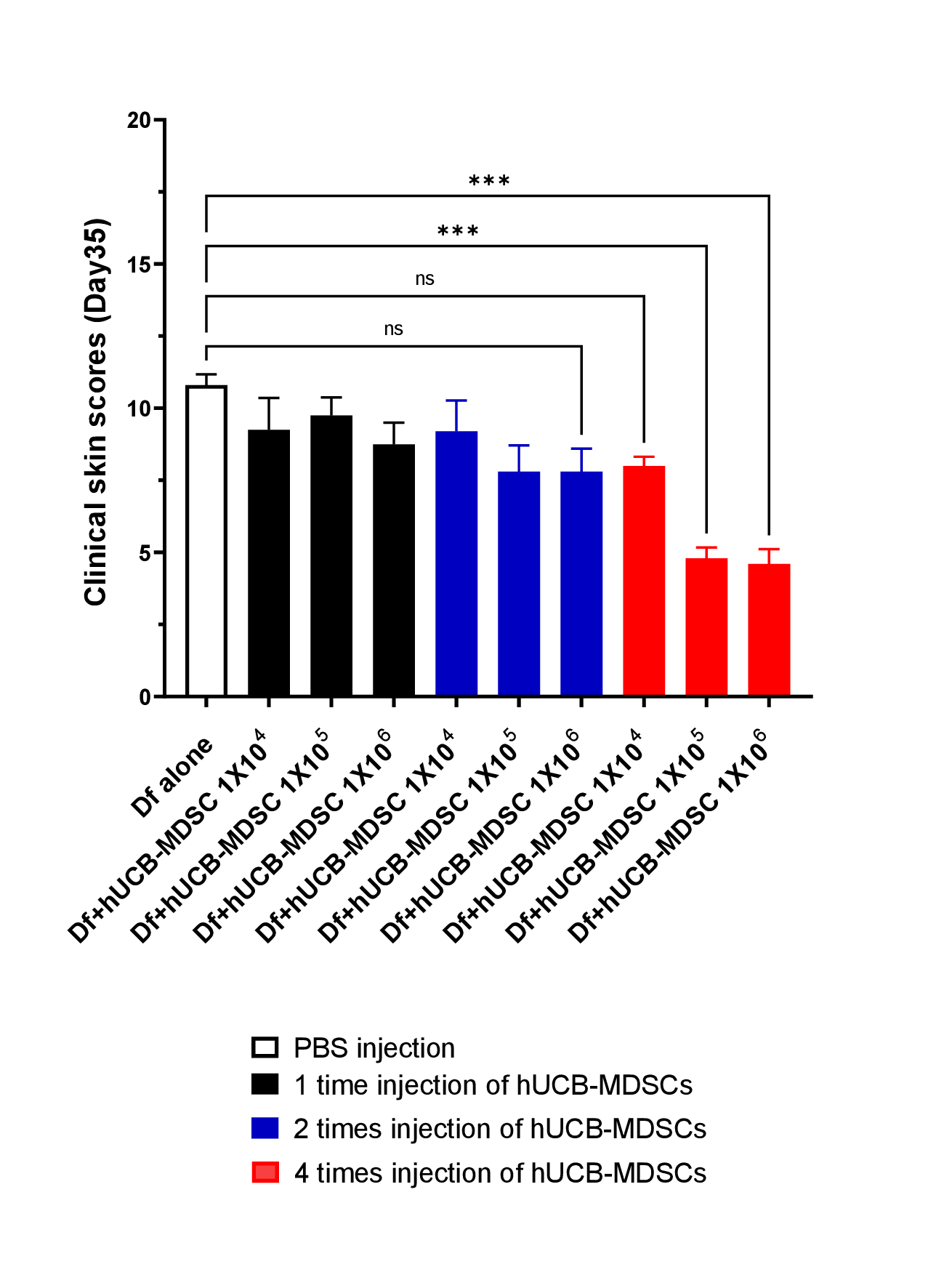

Supplement: Supplementary file 3 [file Image_2.tiff]

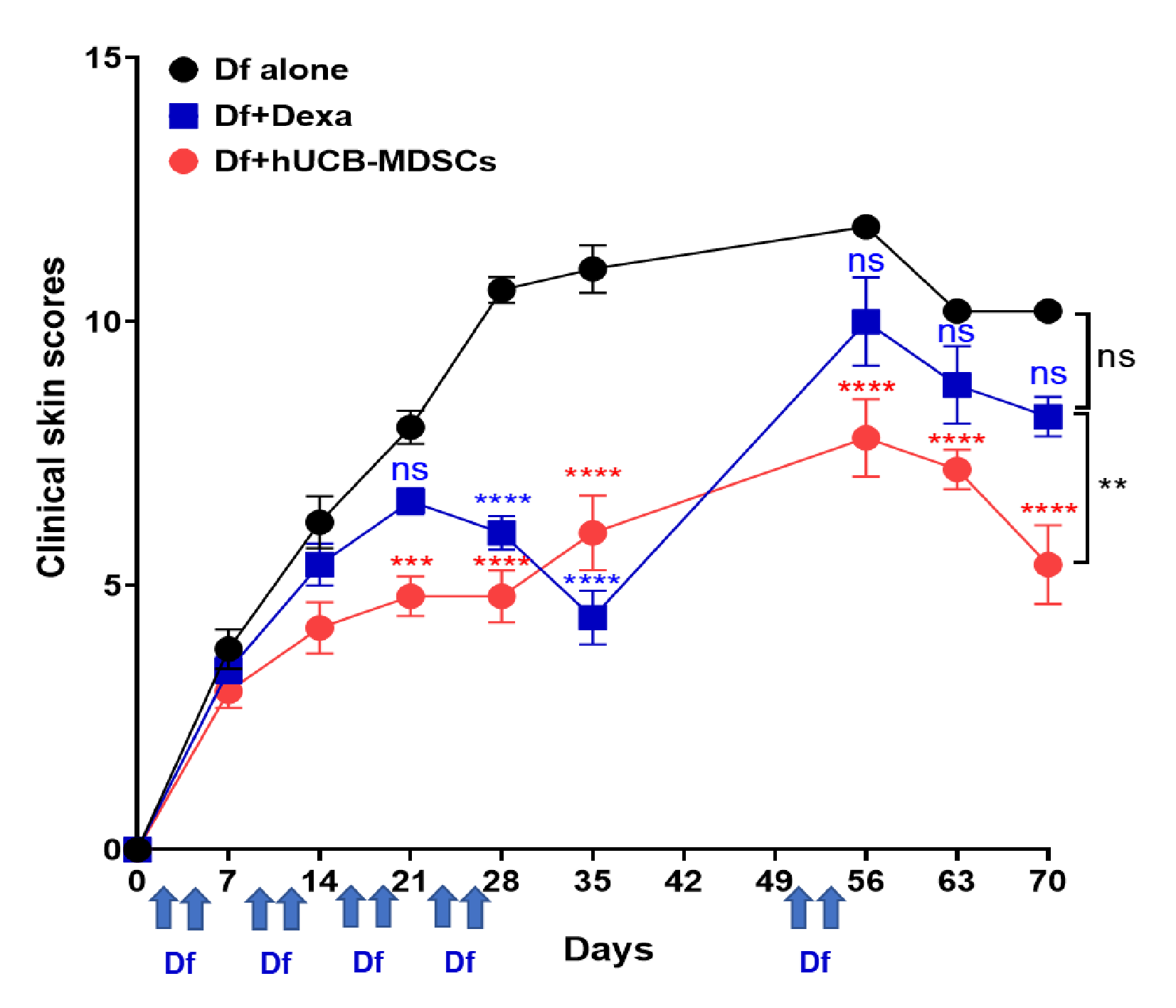

Supplement: Supplementary file 4 [file Image_3.tiff]

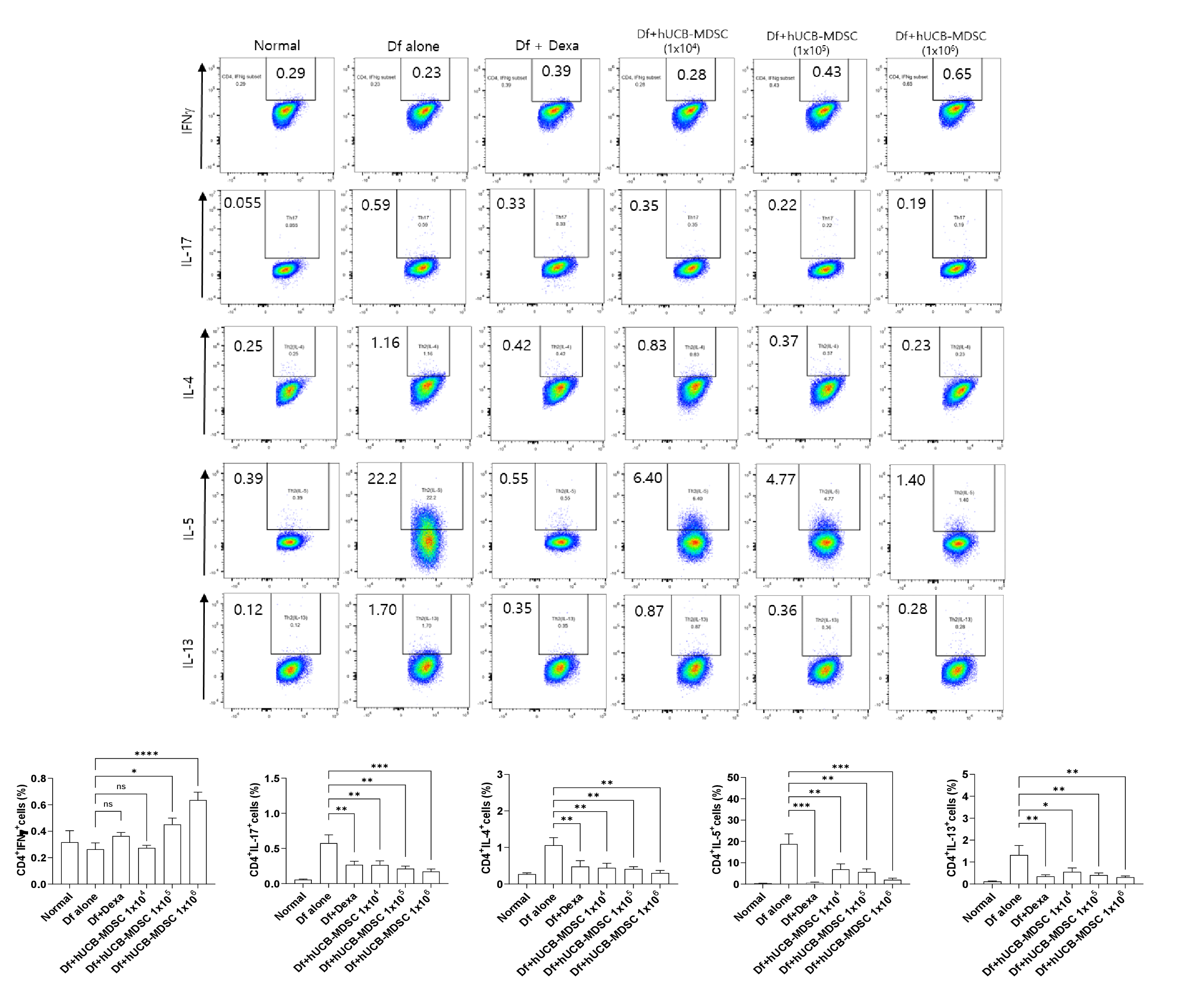

Supplement: Supplementary file 5 [file Image_4.tiff]
